# The Thirty-Seventh Annual Commencement of the Baltimore College of Dental Surgery

**Published:** 1877-04

**Authors:** 


### ARTICLE IV.

*The Thirty-Seventh Annual Commencement of the Baltimore College of Dental Surgery* was held at the Academy of Music, Baltimore City, on Friday evening, March 9th, 1877, and was unusually interesting. Music was furnished by the Independent Greys Orchestra, a number of fine selections from Verdi, Strauss and other composers being rendered.

On the stage were seated some two hundred persons, including the Faculty, Graduates, Alumni and invited guests, among the number dental and medical representatives from all parts of the United States, Canada, and also portions of Europe.

The exercises commenced with prayer, by the Rev. Dr. W. M. Postlethwaite, of the Episcopal Church; after which the Dean, Prof. F. J. S. Gorgas, D. D. S., M. D., announced the Graduates, the authority by which the degree of "Doctor of Dental Surgery" was conferred, and the qualifications necessary.

The degree of "Doctor of Dental Surgery" was then conferred upon the following members of the Graduating Class, all of whom had attended full courses of dental lectures, and been examined on Dental Surgery and Therapeutics, Clinical Dentistry, Dental Mechanism and Metallurgy, Chemistry and Materia Medica, Physiology, Pathology and Anatomy, etc.

| | Subject of Thesis. |
|---|---|
| Pauline Boeck, | *Germany* —Caries of the Teeth. |
| Thomas Washington Crozier, | *Virginia.*—Extraction of Teeth. |
| George Homer Bowman, | *Virginia.*—The Dental Pulp and its Diseases. |
| Elias Drayton Earle, | *Florida.*—Operative Dentistry. |
| Hammett Xavier Gale, | *Ohio.*—The Art of Applying Artificial Teeth. |
| Walter S. Harban, | *Maryland* —The Effects of Diseased Teeth on the System. |
| Sandy Stuart Harris, | *Virginia.*—Caries of the Teeth. |
| Robert Yates Henley, Jr., | *Virginia.*—First Detention. |
| Thomas Huggins, | *England.*—The Fifth Pair of Nerves. |
| Frederick Koerner, | *Maryland.*—Nitrous Oxide Gas. |
| Benjamin Lanier Lane, | *Georgia.*—Circulation of the Blood. |
| John E. La Motte, | *Maryland.*—Dental Caries. |
| Luke Johnson Pearce, | *Maryland.*—Dental Surgery. |
| Charles Hector Reynolds, L. D. S. | *Canada.*—Morbid Effects of Dentition. |
| James Mathews Roberts, | *Maryland.*—Dental Development. |
| James Edwin Shreeve, | *Maryland.*—Comparative Anatomy of the Teeth. |
| Henry Singruen, | *Germany.*—Odontalgia. |
| Alonzo Stouch, | *Pennsylvania.*—Characteristics of the Teeth. |
| Marion William Williams, | *Tenneseee.*—Dental Hygiene. |

The Valedictory Address was delivered by Dr. William H. Dwinelle, of New York City, and was received with marked attention by the large and brilliant audience present. This Address contained pleasant and interesting references to the past thirty-seven years history of the institution and the dental profession, and paid a glowing tribute to the memory of the founders of the Baltimore College of Dental Surgery, and encouragement for the graduates, to whom the speaker recommended patience, perseverance, and constant study.

The Class Address was delivered by George Homer Bowman, of Virginia. His style of delivery was so commendable that the attention of the large audience was attracted and closely held throughout.

The stage was handsomely decorated by the large number of beautiful boquets presented to the graduates, some of whom received more than they could carry.

The Thirty-eighth Annual Session will commence on the 15th of October, 1877, and continue until March, 1878. The Summer Session will commence April 1st, and continue until the last of June, 1877. The Infirmary is open during the entire year for dental operations.

After the close of the exercises at the Academy of Music, a large number of the Alumni, members of the graduating class, and invited guests, among whom were distinguished representatives of other professions, proceeded to the Masonic Temple, where the Alumni Supper was served, which proved a thoroughly enjoyable affair. Dr. S. J. Cockerille, of the class of 1853, President of the Alumni Association, occupied the chair, and very gracefully performed the duties of presiding officer, announcing that this sumptuously elegant repast was tendered to the Alumni by the Faculty of the Baltimore College of Dental Surgery. The regular toasts were read by Prof. Gorgas, and responded to by Dr. Wm. H. Dwinelle, of N. Y., Dr. Jno. Allen, of N. Y., Dr. S. J. Cockerille, of Washington, Dr. George A. Mills, of Baltimore, Profs. Thomas R. Brown, Thomas Opie, John S. Lynch, Thomas S. Latimer, Charles F. Bevan, of the College of Physicians and Surgeons, Prof. Henry R. Noel, M. D., Dr. A. J. Volck, Prof. E. Lloyd Howard, M. D., Prof. J. B. Hodgkin, Dr. T. A. La Far, of Baltimore, and Dr. J. B. Wood, of Richmond, Va., Dr. D. McFarland, of Washington City, Dr. M. W. Williams, of Tennessee, and Dr. C. H. Reynolds, of Canada. All of the speeches were well timed and enthusiastically applauded.

